# Editorial: Biomarkers to Disentangle the Physiological From Pathological Brain Aging

**DOI:** 10.3389/fnagi.2020.00088

**Published:** 2020-04-15

**Authors:** Franca Rosa Guerini, Wee Shiong Lim, Beatrice Arosio

**Affiliations:** ^1^IRCCS Fondazione Don Carlo Gnocchi, Milan, Italy; ^2^Department of Geriatric Medicine, Institute of Geriatrics and Active Ageing, Tan Tock Seng Hospital, Singapore, Singapore; ^3^Geriatric Unit, Fondazione IRCCS Ca' Granda Ospedale Maggiore Policlinico, Milan, Italy; ^4^Department of Clinical Sciences and Community Health, University of Milan, Milan, Italy

**Keywords:** neurodegenerative diseases, aging, biomarkers, bioimage analysis, comorbidities

The worldwide increase of human life expectancy (“lifespan”) along with the concomitant rapid population aging represents the major social phenomena of the last century. This has substantially impacted our societies by virtue of the huge economic implications and public health challenges (Lim et al., 2018). In order to promote healthy aging and further increase the years of life spent without disabilities (“healthspan”) (Beard et al., 2016; Olshansky, 2018), it is necessary to delve deep into the biology of aging and disentangle the mechanisms that underpin the physiological processes from those leading to pathological manifestation.

In this context, dementia as well as others common neurodegenerative diseases are strongly correlated with age. Although advancing age represents the major risk factor for cognitive decline, dementia is not an inevitable consequence of long life, as clearly demonstrated by centenarians who managed to preserve normal cognitive performance despite their age (Arosio et al., 2017). Against this backdrop, the ongoing “biomarker revolution” has been instrumental in transforming the landscape of medical research and practice. The relevance of biomarkers to the field of physiological and pathological brain aging is underscored by two considerations. Firstly, the relative lack of accessibility of brain issue for diagnostic or research purposes, and secondly, the understanding that neurodegenerative diseases are often characterized by a long preclinical phase has fuelled transition toward a biology-grounded framework and definition such as the NIA-AA Research Framework for Alzheimer's disease (AD) (Jack et al., 2018).

However, the available biomarkers currently used for AD generally describe single aspects of the patient's state and are only modestly associated with clinically meaningful manifestations. The combination of biomarkers capable of assessing the “real” biological age of the individual may thus represent a viable strategy to support the identification of disease-specific trajectories and, in parallel, provide insights about the aging phenomenon. The present Research Topic is therefore timely in advancing the ongoing discourse beyond the need of disentangling “physiological” from “pathological” brain aging to deepening existing understanding and offering new vistas for further development in the field.

Using cerebrospinal fluid (CSF) biomarkers for AD as a paradigmatic example, Canevelli et al. discussed the methodological issues and challenges that pertain to four key areas: (1) definition of reference values; (2) identification of reference standards specific for the disease of interest (i.e., AD); (3) proper inclusion and contextualization within the diagnostic process; and (4) statistical processes supporting the whole framework. They concluded that various methodological issues remain to be addressed in order to perform an adequate and complete clinical validation of candidate CSF biomarkers for AD.

Five papers in this Research Topic shed precious insights into emerging biomarkers from readily available biological samples. In their comprehensive review, D'Anca et al. explored the current understanding of role of exosomes in physiological aging and age-related neurodegenerative diseases such as AD, Parkinson's Disease (PD) and frontotemporal dementia. Insights from the study of exosomes and their genetic cargo (such as lipid, proteins, mRNAs, and ncRNAs) have elevated their role beyond mere waste disposal function to fundamental mediators of intercellular communication, akin to the proverbial double-edge sword serving as Trojan horses of neurodegeneration vis-à-vis providing neuroprotection from neurodegeneration. Exosomes can be detected in many biological fluids, enhancing their appeal as potential sources of biomarkers suitable for use in clinical practice.

Similarly, Tay et al. prospectively studied serum levels of Dickkopf-1 (Dkk-1) in older adults with mild cognitive impairment (MCI) and mild-to-moderate AD. The findings revealed that Dkk-1 increased significantly from baseline amongst progressors, while non-progressors exhibited decremental Dkk-1 at 1 year, alluding to the putative role in MCI and AD progression of dysfunctional Wnt signaling through Dkk-1 antagonism. Sun et al. demonstrated that cofilin 2 expression was significantly increased in AD patients and different AD models (animal and cell), with good discriminatory ability that distinguish AD from healthy subjects and in differential diagnosis of AD from vascular dementia. By studying 391 cognitively normal subjects aged 23–91 years from Asia, USA and Europe, Lue et al. characterized the relationship between age and three plasma AD core biomarkers (Aß40, Aß42, and t-Tau), provided the normal ranges of Aß species and t-Tau in plasma, and explicated the development of a dynamic relationship between these biomarkers from middle to old age. Lastly, Falconi et al. investigated the transcriptional regulation of the Adenosine A2A receptors (A2ARs) gene in human peripheral blood mononuclear cells obtained from PD patients and in the striatum of the 6-hydroxydopamine-induced PD mouse model. They reported an increase in A2AR mRNA expression and protein levels in both human cells and mice that is accompanied by histone acetylation and DNA methylation, paving the way for therapeutic interventions in future.

Another prominent theme in this Research Topic was the contribution of advanced neuroimaging biomarkers. For instance, in terms of explicating mechanisms that underpin underlying pathophysiological processes, Wang, Li et al. shed light on the neural mechanisms of working memory deficits in MCI. By combining static and temporal dynamic examination of amplitude of low-frequency fluctuations (ALFF) from functional magnetic resonance imaging, they reported background network changes especially in the parietal and temporal lobes during working memory states in MCI. Similarly, Pur et al. enhanced understanding by demonstrating the moderating effect of cortical thickness on blood oxygen level-dependent (BOLD) signal variability age-related changes and highlight the importance of considering these effects when evaluating BOLD_SD_ alternations across the lifespan. Kobayashi et al. examined whether dementia with Lewy Bodies (DLB) follows an AD-type trajectory whereby amyloid-ß deposition begins considerably before onset of dementia. They observed a low amyloid load in REM sleep behavioral disorder, a prodromal symptom of DLB, suggesting that this phenomenon does not always precede the onset of cognitive decline in DLB. Lastly, through the use of MRI voxel studies to examine neural substrates, Wu, Geng et al. implicated the left-precentral cortex and left inferior frontal gyrus areas that accounted for olfactory impairment in a cohort of AD and MCI patients in the Chinese Han population.

Other papers in this Research Topic illuminated the knowledge gap in our understanding about the role of misfolded protein accumulation in neurodegenerative diseases. The extracellular accumulation of amyloid beta (Aβ) peptide is the paradigmatic example, with recent neuroimaging capabilities in tracking Aβ deposition a fascinating way to distinguish if the observed Aβ accumulation is characteristic of the aging process or conversely, an etio-pathogenetic mechanism of AD (Canevelli et al., 2017). Hatashita et al. examined [18F]-flutemetamol (FMM), a fluorinated derivative of the prototypic [11C]-Pittsburgh Compound B (PIB), and demonstrated that [18F]-FMM PET imaging can track longitudinal changes in Aβ deposition across the AD spectrum, similarly to [11C]-PIB PET. Notably, they reported that the increase in Aβ deposition is not constant across the AD spectrum but faster in the predementia stage (Hatashita et al.). Using dynamic [18F] florbetapir PET, Verfaillie et al. investigated the possibility that self-perceived cognitive decline (SCD), generally associated with a three- to six-fold increased risk of AD, may reflect an early symptom of Aβ related pathology. They concluded that Aβ load was associated with SCD related worries rather than subjective cognitive functioning *per se* (Verfaillie et al.).

Other papers highlight advances in neuroimaging techniques that pave the way for fresh perspectives in early diagnosis of AD. Utilizing sophisticated measurements of cortical thickness with 3.0-Tesla MRI in a large population of cognitively normal individuals and patients with AD continuum, Lee et al. described an age-dependent cortical thinning with relative sparing of the precuneus and inferior temporal regions. In contrast, late-stage amnestic MCI and moderate to severe AD were associated with widespread cortical thinning including the precuneus and inferior temporal regions (Lee et al.). Furthermore, the study by Wu, Xu et al. provides proof-of-concept evidence that a topological examination of the structural connectivity networks with different parcellation schemes can provide important complementary AD-related information and thus contribute to a more accurate and earlier diagnosis of AD. Another promising technique involved amide proton transfer (APT) imaging as an imaging modality to detect tissue protein. In fact, Wang, Chen et al. adopting a modified APT method (APTSAFARI on a 7.0T animal MRI scanner) in animal models, demonstrated that APT imaging could potentially provide molecular biomarkers for non-invasive diagnosis of AD. Using direct non-invasive brain network electrophysiological imaging, Zifman et al. established that this new technique can be used both to monitor brain aging and for early detection of abnormal changes leading to neurodegeneration.

Bioimaging techniques are also useful for the differential diagnosis of PD. Dopamine-transporter SPECT (DAT-SPECT), diffusion tensor imaging (DTI), and structural magnetic resonance imaging (sMRI) provide unique information about neurotransmitters and microstructural properties in PD. The longitudinal study of Lorio et al. described how the combination of these imaging modalities can be used as biomarkers of PD severity and prognosis which can be potentially useful for clinical trials. Likewise, Pelizzari et al. proposed the concomitant use of DTI to detect brain tissue microstructural alterations, together with arterial spin labeling (ASL) MRI to analyse abnormal cerebral perfusion patterns in PD. The study showed that DTI is a more sensitive technique than ASL to detect alterations in the basal ganglia in the early phase of PD, suggesting that a relationship between microstructural integrity and perfusion changes in the caudate may be present (Pelizzari et al.).

Papers in this Research Topic also addressed the issue of age-associated co-morbidities, particularly cardiovascular diseases, and related risk factors such as Type II diabetes mellitus (T2DM), which often confound the measurement and interpretation of neurodegenerative biomarkers. Exploring the association between white matter hyperintensities with higher Intima-media thickness (IMT) and blood pressure variability, Chen et al. developed an innovative predictive model to evaluate white matter burden in hypertensive patients using metrics in 24-h ambulatory BP monitoring (systolic blood pressure [SBP] and daytime SBP standard deviation) and carotid ultrasound IMT. Leveraging upon the diagnostic potential of diabetic retinopathy (DR) afforded by its time-course which precedes the occurrence of T2DM cognitive impairment, Lu et al. verified the correlation between DR from fundus examination and T2DM cognitive impairment. They further established using magnetic resonance spectroscopy (^1^H-MRS) that this may be attributed to bilateral changes in hippocampal brain metabolism, alluding to the potential role of ^1^H-MRS for early diagnosis of T2DM cognitive impairment (Lu et al.).

A major pathogenetic influence of co-morbidities or their treatment that have emerged in recent years is that of concomitant inflammatory processes that may alter disease manifestation and confound biomarker interpretation. An example is the cognitive decline observed in some HIV subjects during ART-mediated viral suppression, a phenomena which has been ascribed to cytokine-mediated inflammation. Bandera et al. suggested an omics approach (transcriptomics, proteomics, and metabolics) to accelerate the discovery of reliable biomarkers of HIV-associated neurocognitive disorders in the current era of virological suppression with modern anti-retroviral therapy. Similarly, persistent inflammation has been implicated as a cardinal mechanism of neurocognitive impairment and neurodegenerative features in Multiple Sclerosis (MS). Jakimovski et al. reported the novel finding that almost half the elderly subjects with MS are impaired on tests of cognitive processing speed or verbal fluency. Since the deficit in verbal fluency is not a typical hallmark of the cognitive profile associated with MS, it may represent a unique trait of old persons with MS neurocognitive profile (Jakimovski et al.).

Lastly, there is growing interest in the role of physical activity and exercise as “medicine” that can be prescribed for brain health. In their comprehensive review, Barha et al. underlined the role of biological sex as a potential moderator in the relationship between physical activity and brain health in older adults. They suggested that different neurobiological mechanisms (e.g., neurotrophic factors, neuroplastic processes, hormones, and neurotransmitter systems) as well as physiological adaptations to physical activities could be responsible for the differences in trajectories of decline observed in men and women (Barha et al.). This highlights the importance of taking into consideration the potent moderating influence of sex differences for both cognitive and neural outcomes in future therapeutic and rehabilitative interventions involving physical activity in older adults.

As guest editors for this Research Topic, we commend this collection of 23 articles to our readers as a timely addition and important contribution to the field. We are confident that this will spur further discourse and open avenues for further research into the rapidly evolving and fascinating area of biomarkers to disentangle physiological from pathological brain aging.

## Author Contributions

FG, WL, and BA conceived the manuscript. FG and WL drafted the paper. BA critically appraised and edited the manuscript. All authors read and approved the final version of the paper.

### Conflict of Interest

The authors declare that the research was conducted in the absence of any commercial or financial relationships that could be construed as a potential conflict of interest.
